# Identifying Palliative Care Needs in Assisted Living

**DOI:** 10.1093/geroni/igaa057.2854

**Published:** 2020-12-16

**Authors:** Daniel David, Abraham Brody, Tina Sadarangani, Bei Wu, Tara Cortez

**Affiliations:** 1 New York University, New York, New York, United States; 2 NYU Rory Meyers College of Nursing, New York, New York, United States; 3 NYU, New York, New York, United States

## Abstract

Many residents of Assisted Living (AL) confront serious illness and therefore might benefit from greater access to Palliative Care Services to improve quality of life. We surveyed resident records and AL nursing staff to identify patients in need of Palliative Care. Preliminary findings showed that nurses predicted 23% would not be alive and 49% would no longer live in AL. A majority of residents were over the age of 90, yet 30% did not have a reported code status. These findings suggest that a substantial portion of AL residents may have unmet needs with respect to palliative care. Future interventions are needed to support advance care planning conversations and make palliative care more accessible to this population.

